# Flesh Without Blood: The Public Health Benefits of Lab‐Grown Meat

**DOI:** 10.1007/s11673-023-10254-7

**Published:** 2023-09-01

**Authors:** Jonny Anomaly, Heather Browning, Diana Fleischman, Walter Veit

**Affiliations:** 1 Centro de Estudios de Filosofía, Política y Economía, Quito, Ecuador; 2 University of Southampton, Southampton, UK; 3 University of New Mexico, Albuquerque, NM USA; 4 University of Bristol, Bristol, UK

**Keywords:** Clean meat, Animal welfare, Antibiotic resistance, Zoonotic disease, Synthetic meat

## Abstract

Synthetic meat made from animal cells will transform how we eat. It will reduce suffering by eliminating the need to raise and slaughter animals. But it will also have big public health benefits if it becomes widely consumed. In this paper, we discuss how “clean meat” can reduce the risks associated with intensive animal farming, including antibiotic resistance, environmental pollution, and zoonotic viral diseases like influenza and coronavirus. Since the most common objection to clean meat is that some people find it “disgusting” or “unnatural,” we explore the psychology of disgust to find possible counter-measures. We argue that the public health benefits of clean meat give us strong moral reasons to promote its development and consumption in a way that the public is likely to support. We end by depicting the change from farmed animals to clean meat as a collective action problem and suggest that social norms rather than coercive laws should be employed to solve the problem.

## Introduction

Our Paleolithic ancestors relied on a more or less steady supply of protein in the form of meat.^1^ It is now possible to substitute a carnivorous diet with protein derived from plants, but it can be tricky to design a plant-based diet that contains the complete range of amino acids and minerals needed for a healthy human life. Consuming meat from slaughtered animals is still the easiest and often the cheapest way for many to meet their dietary needs and taste for traditional animal meat.

One new development, however, may radically change animal agriculture forever: “clean meat.” Along with new plant-based products that mimic the texture and taste of meat, biomedical engineers can now take either stem cells, or adult animal cells, and induce them to replicate until they become a slice of meat. Creating a steak, with its intricate marbling, is challenging. Creating ground beef or chicken nuggets is more straightforward. So straightforward that in 2020 Israel opened the first clean meat restaurant in human history. Offerings are still quite limited, but the restaurant’s meat suppliers provide proof that some forms of clean meat can be made affordable. Enhancing the nutritional profile of meat is likely to be as feasible as enhancing its taste and texture, though it is still an open question whether this process can scale in a way that would make complex cuts of meat—as opposed to ground beef or uniform slabs of chicken—affordable to everyone.^2^

## Public Health and Animal Agriculture

Despite the increase of vegans and vegetarians (in Western countries), meat consumption continues to grow around the world, and not just because of population growth. Wealthier people tend to eat more meat (Ritchie, Rosado, and Roser 2019). For example, in China the average person in 2010 ate 55kg of meat or four times as much meat as they did in the 1970s (Herzog 2010). And although a small percent of people in the most prosperous countries in Europe and North America have turned to vegetarian diets, most are consuming more meat than ever. Some question whether we can ever justify killing animals for consumption, while others worry more about the *ways* in which animals on factory farms are raised and slaughtered (Singer 2011, chapter 3). But unless a remarkable change of dietary norms occurs, or clean meat becomes widely consumed, it is likely that intensive animal farms will grow in the present century, especially in third world countries. One of the major problems with the growth of factory farms is an increased risk of antibiotic resistance and, more importantly, zoonotic diseases, which occur when non-human animals transfer pathogens like influenza and coronavirus to people (Anomaly 2015, 2020).

Animals raised in the cramped conditions of factory farms are often given a steady dose of antibiotics to avoid disease and promote growth. This gives rise to antibiotic resistant bacteria, which make their way into our general microbial environment, including human households and hospitals (Marshall and Levy 2011). Although it is difficult to quantify with precision, experts estimate that about half of all antibiotics in the world are given to farm animals (O’Neill 2015). If animals were given more roaming space, they wouldn’t need antibiotics. But it is often more profitable to raise animals in factory-like conditions, ignoring the cruelty and suffering imposed on them and public health costs imposed on people. While European and other Western nations have begun regulating and discouraging the use agricultural antibiotics, their use is skyrocketing in places like China, India, and North Africa (Tiseo, et al. 2020).

A recent example of antibiotic resistant strains of bacteria arising on factory farms and spreading to people occurred in China. Samples of bacteria on intensive animal farms in China show a spike in resistance to colistin, which is a powerful, last-resort antibiotic important for human health (Liu, et al. 2016;l Xu, et al. 2018). This is one of many strains of antibiotic resistant bacteria that have either arisen from, or have been exacerbated by, the use of antibiotics in animal agriculture (Rinsky, et al. 2013; Cuny, Wieler, and Witte 2015; Spellberg, et al. 2016).

Even if we were to end the use of antibiotics in animals, zoonotic diseases are an intrinsic risk associated with animal agriculture. Factory farming only intensifies this risk. There are several ways deadly microbes are transmitted from non-human animals to humans (Greger 2007). One is from consuming animal meat, especially if it is raw or undercooked. Another is by humans changing their ecosystems in ways that expose them to environments that contain deadly pathogens. For example, malaria is transmitted by mosquitoes that breed near stagnant water that humans might use as a source of drinking water for themselves or their farm animals. Lyme disease and plague are contracted from rodents that feed off the scraps of food they find in densely populated human settlements. Domesticated animals living in densely packed environments near people can transmit diseases to us even if we don’t eat them.

As Dorothy Crawford documents, the agricultural revolution improved food security and social stability but dramatically increased infectious diseases in human populations:

For the first time people were living in close contact with herd animals—drinking their milk, butchering and eating their flesh, curing their skins, caring for their young and sick, and sharing their shelter. Many animal microbes took the opportunity to jump ship, finding a new niche in a virgin human population … There is now no doubt that most of the microbes that cause the classic acute childhood infectious diseases, such as smallpox, measles, mumps, diphtheria, whooping cough and scarlet fever, were originally exclusively animal pathogens that at some time in the past crossed the species barrier to infect humans. Today they only infect humans but their DNA sequences contain the tell-tale signs of their past lives. Their closest relatives are among the microbes of domestic animals, and in some cases the molecular clock even pinpoints the timing of their transfer to the early farming era. (2018, 60)

While zoonotic diseases have been afflicting us for a long time, factory farming creates conditions that intensify the risks of novel forms of these diseases (Crawford 2000). Antibiotic resistant bacteria and zoonotic viruses that spread from domesticated animals to humans impose invisible costs on people around the world that are not included in the purchase price of meat. Animal farming also requires more space (often obtained by cutting down forests) and more energy inputs than the production of clean meat (Tuomisto and de Mattos 2011). By contrast, clean meat will allow us to enjoy the taste of traditional meat from animals without imposing health costs on other people and cruelty on animals (Fleischman 2021).

## Animal Suffering

Along with concerns for human and environmental health, there are of course those for the animals themselves. Indeed, perhaps the strongest driving factor for a reduction in the consumption of animal products has been a concern for the welfare of animals used in agriculture, particularly intensively managed or “factory farmed” animals. Many modern farming practices create suffering through husbandry and slaughter practices. Awareness of the welfare problems associated with factory farming was first raised by Ruth Harrison in her 1964 book *Animal Machines*, issues that were further brought to popular attention by Peter Singer in 1975 with the publication of *Animal Liberation*. These books, and others since, have highlighted the range of ways in which intensive farming causes suffering to animals: broiler chickens spending their life indoors with no natural light and less than a square foot each, their beaks partially removed with hot blades to decrease aggression in these crowded conditions, and suffering deformities and lameness from overly rapid growth; sows kept in tiny stalls that don’t permit them to turn around or provide a soft place to sleep, with limited cognitive and behavioural opportunities provided for them to exercise their physical and cognitive needs (Gruen 2011; Harrison 1964; Singer 1975). With over 70 billion animals^3^ farmed annually for human food production, and around another 1–3 trillion fish^4^ caught per year, this is a considerable amount of suffering.

Apart from the suffering caused by improper housing, there are welfare problems with slaughter. Transporting, processing, and killing farm animals are often sources of pain, discomfort, and suffering, with even the best-run slaughterhouses facing failure rates in stunning, leading to millions of animals being processed while still conscious (Lamey 2019). Even if some slaughterhouses try to minimize these negative effects for animals, little if any attention is given to positive experiences. The mere absence of pain is not the same as a *good* life. There is also the moral concern raised by the very fact of slaughter itself, which on many accounts of animal welfare, will reduce welfare through the shortening of life and removal of future opportunities for pleasure (Browning and Veit 2020).

Utilitarians are especially concerned with minimizing suffering. But other ethical views emphasize the rights of animals, including the freedom not to be used for human ends without sufficient justification (e.g. Regan 1983). The very acts of farming and slaughtering animals—especially in intensive farming operations—are acts of cruelty towards animals, over and above the suffering caused. Across a range of views, as well as public opinion, it is generally accepted that animals possess at least some moral standing and, all else being equal, it is important to consider their welfare. This is particularly true for captive animals, where humans are entirely responsible for the conditions and quality of lives of the animals in their care.

The reason these issues raise moral concerns is because of the *experience* of animals. Many animals—including all the vertebrate animals used in agriculture—are sentient, meaning they are capable of experiencing positively and negatively valanced mental states such as pleasure and pain. It is this experience that grounds our moral concern for their treatment (Browning 2019). This was famously and eloquently proclaimed by Jeremy Bentham: “The question is not, Can they *reason*? nor, Can they *talk*? but, Can they *suffer*?” (Bentham 1879, 309). This concern for the experience of animals can be seen throughout a range of ethical views, such as the utilitarianism of Singer (1975), the interest-based rights views that ground interests in the capacity for pleasure and suffering (e.g. Beauchamp 2011; Cochrane 2012; Gruen 2011) and even some virtue ethicists, who see recognition of sentience as giving rise to virtues such as compassion and respect in our interactions with them (Hursthouse 2011). It is also a strong focus of much of the work in animal welfare science (e.g. Dawkins 1980; Fraser 1999; Mellor et al. 2020). The general claim is that having the capacity for first-person experience makes sentience morally significant: “It is the fact that sentient beings care about how their lives go that generates a distinctive moral claim on us” (Donaldson and Kymlicka 2011, 33).

Any animal that is sentient is capable of suffering and this gives us moral reason to want to minimize suffering. As philosophers such as Nietzsche emphasize, suffering is not always bad. Sometimes we deserve to suffer, and sometimes the suffering of some has compensating benefits for others. Nevertheless, animal suffering has little if any meaning: it does not resemble the kind of suffering we associate with a tortured artist such as van Gogh, which is compensated for by the sublime beauty of his art. Animal suffering has no deeper meaning. It does not occur in the context of generating lasting artwork or scientific discovery. And if we can get the benefits of eating animal meat without imposing the costs on raising and killing farmed animals, this seems like an unequivocal moral good.

Whichever ethical views one holds, it is clear that intensive animal farming results in a large amount of suffering that would be prevented by the consumption of clean meat. Although there are alternative systems of meat production—often called “humane farms”—that cause less suffering, these are typically considered unsustainable for affordable production of animal products at the current scale of consumption (Singer 2011). By contrast, lab-grown meat is a simple insentient tissue, incapable of suffering and should thus not raise any moral concerns. Use of lab-grown meat in place of animal agriculture could significantly reduce, if not eliminate, the amount of suffering caused through food production. Since general ethical arguments against clean meat have been reviewed and (we think) refuted elsewhere (Schaefer and Savulescu 2014), we focus the rest of the paper on psychological and behavioural barriers to its uptake.

## Disgust

Despite the obvious benefits of clean meat, many people find the idea of eating cultivated meat disgusting. This is important to the future of clean meat because disgust has a big influence on what we consume. The main reason disgust seems to have evolved is that it helps us to distinguish and avoid foods that are unsafe to eat, what Rozin calls “the omnivore’s dilemma” (1976). The idea is that humans are unique in constantly seeking out new forms of food, some of which is bound to be pathogenic, even deadly. Meat is more likely to contain harmful pathogens than plants, meat is responsible for more foodborne illnesses than plants, and societies have far more food taboos with regard to meat than other foods (Fessler and Navarrete, 2003). Moreover, some of these taboos are beneficial.

Some food-based taboos and aversions are based in our evolved biology: they are built into us (Curtis, et al. 2011). For example, the smell of rotting carcasses makes us turn away in disgust, while it attracts vultures to a fresh kill. This makes perfect sense since our stomachs are poorly adapted to digest the kinds of pathogens that vultures can contend with. But even our biological predispositions can be altered, especially when they are directed by social norms that shape our behaviour more substantially than smell and sight.

As Joseph Henrich has argued, social norms can embody a kind of implicit cultural knowledge acquired through trial and error (2015). To take an especially interesting example, when the Spanish conquistadors came to the Americas, they quickly took up the habit of eating corn, like the Mayans they conquered. According to Henrich, native Americans had learned to soak corn with a mixture of burnt shells, or ashes from fire, before cooking and eating it. This apparently allowed the corn to release essential vitamins. When Spaniards decided to skip the seemingly strange process and go straight to eating the corn, they developed sometimes deadly vitamin deficiencies. This illustrates how our willingness to eat new foods can be beneficial but sometimes only if it’s paired with social taboos against certain practices—in this case, against eating corn without going through the proper rituals. From an evolutionary standpoint, disgust toward novel foods makes sense, as does a disposition to eat novel foods only after performing the right rituals or following the predominant social norms.

Food preferences form and crystallize at an early age and mostly reflect the foods we are exposed to (Birch 1999). Most importantly for clean meat, food neophobia—the aversion to unfamiliar foods—is greater for animal-based foods than plant-based foods (Çınar, Karinen, and Tybur 2021). This almost certainly reflects the fact that many of humanity’s deadliest pathogens have come from eating animals rather than plants (Greger 2007). Thus, it is not surprising that more familiar conventional meat production is generally considered more palatable and less disgusting to the average person than the alternative, since no one grew up with this kind of food (Siegrist and Hartmann 2020). It is likely that this consideration was part of the branding of cultured meat as “clean meat” rather than “lab meat” or “in vitro meat,” which it is sometimes called.

A few studies have investigated consumer acceptance of clean meat. In one survey of American adults, 65 per cent were willing to try clean meat and about one-third said they would be willing to eat clean meat as a replacement for farmed meat (Wilks and Phillips 2017). Men, who tend to eat more meat than women, also had a more positive view of clean meat in this American sample. In a sample of over three thousand participants from the United States, India, and China, 93 per cent of Chinese participants said they were likely to purchase clean meat, as were 86 per cent of Indian participants and 75 per cent of American participants (Bryant and Barnett 2020). In keeping with ideas about sex differences and food aversions, men and those who are less disgust-sensitive are more favourable towards clean meat (Bryant and Barnett 2020).

If clean meat is going to become an important solution to the many problems caused by the global animal industry we must consider how to address the prudential problems of disgust. One of the main obstacles to producing clean meat is managing contamination, since the animal cells that comprise clean meat do not have a functioning immune system. Any contamination of clean meat on the market or a recall could have long-term effects on attitudes to clean meat. This could mean that people will continue to buy more familiar meat, for example from CAFOs, for decades, unless we can maintain high sanitation standards for synthetic meat.

People are often more concerned with what’s delicious than what is virtuous (Bryant and Barnett 2018). That’s why making sure that clean meat is both safer *and tastier* than conventional meat can go a long way to inducing ordinary people to want to eat it. Still, we think that the rise of zoonotic diseases associated with conventional meat production can turn the tide of disgust against forms of animal agriculture that can cause pandemics, and make clean meat more psychologically palatable. Future marketing campaigns may even be able to make use of video material of the disgusting features of large-scale industrial animal farming, which is not at all appetizing to most people, and compare it to the clean and futuristic looking synthetic meat labs cropping up in places like Holland and Israel. That is, by highlighting disgusting features of farmed meat, we may be able to reduce the visceral revulsion some may have toward synthetic meat. Part of our efforts must be directed against an anti-scientific attitude toward “artificial” foods. “Natural” does not automatically mean good, and highlighting how cruel large-scale industrial farming is toward animals, along with the public health risks it poses to people, may help to reduce the stigma against clean meat.

## Collective Action

So far, we have argued that there are good reasons to consume clean meat in order to promote public health and reduce animal cruelty. But whereas animal suffering can plausibly be reduced with any person choosing to forego meat from slaughterhouses for “bloodless” clean meat, the public health benefits associated with clean meat are largely a function of *how many* people make the switch away from animal consumption and toward either clean meat or plant-based protein.^5^ Whether such a significant threshold will be reached is questionable under current conditions, since there may be some reluctance to switch to clean meat to the extent that the benefits of consumption accrue mostly to other people, while the costs are borne by the individual consumer. The costs might be monetary (until the price of clean meat falls) or aesthetic (to the extent that the taste or texture differs from traditional meat). In other words, for those who are not already excited by the prospect of eating clean meat, the shift from consuming traditional meat to clean meat may generate a collective action problem deserving of public attention.

Collective action problems occur when there are benefits to a group from each of their members contributing to some collective good but when there are incentives for each to refrain from contributing. They are close cousins of “public goods” problems or “commons tragedies” in economics and are often modelled by game theorists as multi-player prisoner’s dilemmas or assurance games. Which model we use to capture a collective action problem depends on the motivations of the people we’re modelling and on what we think the collective benefits and costs of their actions are likely to be.

If clean meat scales in a way that it becomes as cheap and healthy as “natural” meat, and if it also has collective health and environmental benefits, we might expect everyone to change their eating habits and consume it. But this is optimistic scenario may not come to pass. First, as discussed above, many people might need to overcome resistance to what they regard as “artificial” food. We think this is likely to happen as clean meat becomes a cheap alternative, is understood to be healthy, and social norms change. But the second reason people might resist clean meat even if the collective benefits are large is that we often ignore the external costs or benefits of our consumption habits. When the consequences of our actions are borne by other people, we often fail to include them in the implicit cost-benefit analysis each of us does before we act. For example, when we think about which road to drive on in order to get home quickly, we fail to include the cost we impose on others by *increasing* congestion on one road and *decreasing* it on other roads. When each of us discounts these external effects, it makes little difference. But when all of us do so, the aggregate effect is big—it is the difference between heavy traffic and light traffic.

In the case of traffic, it’s hard to know what the total effects of our decisions are likely to be.

So it’s both rational and morally excusable to ignore these effects. However, in the case of other aggregate harms, like the air pollution we cause when we use certain sources of energy, or the public health risks we impose when we consume meat from factory farms, the consequences of our choices are a little clearer. It’s true that in any given case of contributing to a public health problem like air pollution, we are unlikely to impose any discrete harms on other people. But it’s also true that in cases like this we are imposing probabilistic harms on others, especially when many other people make the same choices.

Our moral psychology evolved to solve small-scale collective action problems. Our ancestors constantly faced situations in which they could contribute to a collective good, such as banding together to hunt large animals or free ride on the efforts of others. Moral emotions like shame and guilt for violating pro-social norms, and esteem and pride for following pro-social norms, facilitate the emergence and stability of cooperation (Bowles and Gintis 2013). But our moral psychology tends to be more effective in getting small groups to cooperate, especially when the harms from not cooperating are immediate and easy to detect. But when harms are invisible, diffuse, and probabilistic, people tend to ignore them. Worse still, people are perfectly *rational* (in the economic sense) to ignore such harms.

To the extent that rationality involves using our time and energy to efficiently satisfy our goals, it makes little sense for ordinary people to try to figure out the total consequences of their actions—whether they’re contributing imperceptibly to traffic on the freeway or pollution in the air or public health risks like antibiotic resistance. It’s virtually impossible for us to calculate the external harms and benefits of our actions, and in many cases we are unlikely to make a significant difference to a cumulative outcome through our individual actions. For example, if I buy a single slab of roast beef from a butchered cow raised on a factory farm, and thereby slightly increase demand for factory farmed meat, my individual action is unlikely to impose public health risks on other people. Because most people implicitly understand this, so many don’t investigate the connection between eating factory farmed animal meat and zoonotic viruses or antibiotic resistant bacteria, even if many people are disturbed the cruelty involved in factory farming.

Many Americans think that they should consume less meat, in part because of the cruelty they associate with factory farms. According to a recent survey, 49 per cent of U.S. adults supported a ban on factory farming, and 54 per cent said they are trying to consume less meat, dairy, and eggs.^6^ If consumer behaviour was consistent with these sentiments, we would expect a large proportion of the population to boycott animal products. But the reality is very different, perhaps because many understand that their personal responsibility for the aggregate harms of factory farming is relatively low. Indeed, protein-rich alternatives to meat are a niche product, not especially popular outside of affluent parts of rich countries, despite their low price. This could suggest people will also resist consuming clean meat, despite it having a similar taste and the same amino acid profile as meat from farmed animals.

Moreover, if, as some evidence shows (Herzog 2010), many who label themselves vegetarian are just avoiding red meat for health reasons, it doesn’t seem like the costs associated with factory farming will be solved by the individual actions of consumers. For this reason, we think it is important to focus on how to harness social norms, information, and marketing to help nudge people from the individually rational to the collectively beneficial action.

When influential people publicly change their actions and attitudes, this can create preference cascades that nudge ordinary people from reluctant to willing consumers. We do not advocate psychological manipulation to change people’s behaviour, least of all manipulation that comes from government actors, who are especially prone to abusing their power. But we do want to highlight that most people do not change their minds about how they eat or dress or act by carefully reviewing the arguments and then forging their own path. The psychological literature is clear that most people copy the behaviour of those they admire, or whose word they consider authoritative. In other words, they copy the behaviour of elites, or “norm entrepreneurs” (Ellickson 2001).^7^ This insight about psychology can be used for malevolent ends. History is replete with examples of malevolent tyrants or mundane bureaucrats who use their intuitive understanding of human psychology to achieve their own private ends. But it is perfectly reasonable—even praiseworthy—for influential elites to try to shape public opinion when they have strong reasons to believe that reluctance to adopt a new but socially beneficial norm has resulted from an unjustifiable prejudice. We think this is clearly the case when it comes to reluctance to consume clean meat.

## Conclusion

In this article, we have tried to make a concise case for clean meat by highlighting the immense animal suffering that occurs on factory farms, and the public health consequences of intensive animal farming. Recent pandemics have made us painfully aware of the potential dangers of diseases “jumping hosts” between animals and humans. We think it is imperative for animal advocacy organizations and public health advocates to support research into making clean meat significantly cheaper and more widely available. Nevertheless, we have also acknowledged that resistance to radically new kinds of food poses a psychological barrier that will need to be overcome for clean meat to fulfil its potential as one of the single greatest triumphs of the human species in reducing the suffering of animals, and people.

## Footnotes

1 Some even credit increased meat consumption with the rapid evolution of large brains (Aiello and Wheeler 1995).

2 For an overview of the scientific challenges of creating clean meat, see Ben-Arye and Levenberg (2019). For a less technical overview, see Bombardner’s (2018) article or Paul Shapiro’s (2018) book-length account of the subject.

3 World Animal Protection. 2023. Animals in farming [webpage]. https://www.worldanimalprotection.org.au/our-work/animals-farming-supporting-70-billion-animals. Accessed June 29, 2023.

4 Fishcount. 2019. Fish count estimates. http://fishcount.org.uk/fish-count-estimates-2. Accessed June 29, 2023.

5 The threshold point does not apply to animal suffering, which is bad even if it is rare, and the badness of which is scalar rather than lumpy or binary.

6 https://www.sentienceinstitute.org/animal-farmingattitudes-survey-2017.

7 Of course, we recognize that “elites” often has a bad connotation because of the composition of the current ruling class in the West. But human history and psychology suggests that the emergence and influence of elites is inevitable (Burnham 2020). It is only a question of who fills that description in a particular time and place.
